# Therapeutic Effect of Low-Level Laser Therapy in Patients with Temporomandibular Disorder

**DOI:** 10.3390/life16081305

**Published:** 2026-08-09

**Authors:** Igor Djordjević, Ana Miletić, Filip Ivanjac, Danica Popović Antić, Minja Miličić Lazić, Vitomir Konstantinović

**Affiliations:** 1 Department for Prosthodontics, School of Dental Medicine, University of Belgrade, 11000 Belgrade, Serbia; igor.djordjevic@stomf.bg.ac.rs (I.D.); anamiletic.eq@gmail.com (A.M.); danica.popovic@stomf.bg.ac.rs (D.P.A.); minja.milicic@stomf.bg.ac.rs (M.M.L.); 2 Department for Maxillofacial Surgery, School of Dental Medicine, University of Belgrade, 11000 Belgrade, Serbia; v.konstantinovic@stomf.bg.ac.rs

**Keywords:** low-level laser therapy (LLLT), temporomandibular disorders (TMD), temporomandibular joint (TMJ), facial pain, craniomandibular disorders

## Abstract

Background: Temporomandibular disorders (TMD) are associated with musculoskeletal pain affecting the masticatory muscles and temporomandibular joint (TMJ). Low-level laser therapy (LLLT) is a therapeutic modality with analgesic, anti-inflammatory, and biostimulatory effects. Protocol and laser parameter settings for TMD management have not yet been established. The aim of this prospective study was to evaluate the effectiveness of low-level laser therapy in reducing pain in TMD patients. Methods: Sixty-three participants treated at the Department of Prosthodontics, School of Dental Medicine, University of Belgrade, were sorted to the LLLT group and the control splint-medication group (SMG), which received occlusal splint therapy, with nonsteroidal anti-inflammatory drug treatment (NSAID; Ibuprofen). Results: Both therapeutic approaches resulted in significant pain reduction and improvement in patients’ quality of life. No statistically significant differences were observed between the groups, LLLT patients demonstrated a higher rate of successful treatment outcomes, reduction in pain intensity during the treatment. Conclusion: The applied LLLT was associated with significant reduction in pain intensity and improvement in oral health-related quality of life in patients with TMD. Although no statistically significant differences between the groups were demonstrated at the individual post-treatment assessment points, these findings support LLLT as a potentially useful non-invasive conservative treatment option.

## 1. Introduction

The term temporomandibular disorder (TMD) encompasses a group of conditions affecting the temporomandibular joint (TMJ), masticatory muscles, and associated structures. Epidemiological studies indicate that 50–75% of individuals experience at least one sign of dysfunction of the masticatory or orofacial system during their lifetime. The orofacial system (OFS) comprises a complex set of organs and tissues responsible for phonation, facial aesthetics, mastication, swallowing, respiration, and sucking. Its principal components include the teeth and periodontium, temporomandibular joints, muscles and ligaments, maxilla and mandible, lips, cheeks, tongue, blood vessels, and nerves. Disturbances affecting any of these structures may impair biological regulation, ultimately leading to dysfunction of the orofacial system once adaptive capacities are exceeded.

Temporomandibular disorders occur most frequently between the ages of 20 and 40 years. The most common signs and symptoms include pain in the region of the masseter and/or temporomandibular joint, pain in the temporal muscle region, headache, ear pain, limited mouth opening, and joint sounds such as clicking and crepitation. The prevalence of painful TMD in the general population ranges from 5% to 12% and is associated with pain, functional limitations, and a reduced quality of life [1,2,3].

Orofacial pain is one of the most common manifestations of TMD and may also be present in conditions characterized by atypical facial pain. Contemporary therapeutic approach promotes a multidisciplinary management aimed not only at alleviating pain but also at addressing the underlying etiological factors contributing to dysfunction [4].

**Figure 1:**
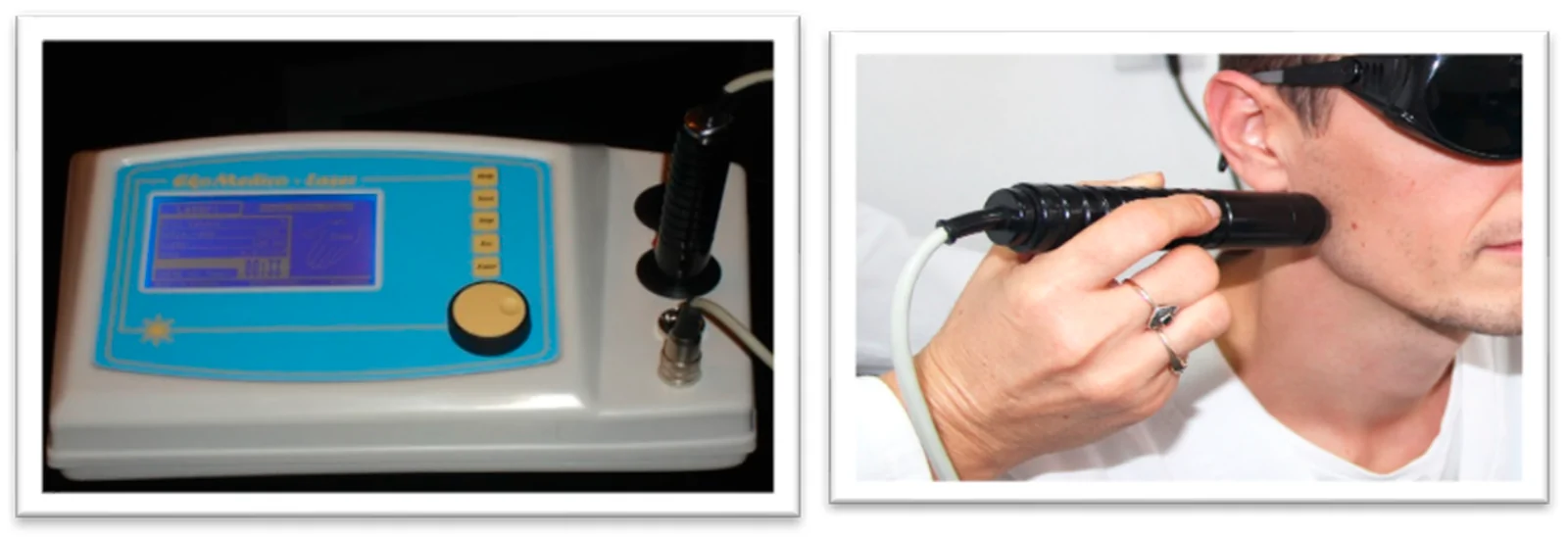
Low-level laser therapy device.

Low-level laser therapy (LLLT) has been shown to be effective in reducing both acute and chronic pain in patients with TMD. Several mechanisms have been proposed to explain the therapeutic effects of low-power lasers (Figure 1). LLLT stimulates the release of endogenous opioids, particularly β-endorphins, increases the pain threshold by modulating cell membrane potential, promotes vasodilation, and exerts anti-inflammatory effects by reducing the levels of prostaglandins, cyclooxygenases (COX-1 and COX-2), tumor necrosis factor-alpha (TNF-α), and transforming growth factor-beta (TGF-β). In addition, LLLT exhibits biostimulatory properties by promoting angiogenesis, stimulating the release of growth factors, enhancing collagen synthesis within the extracellular matrix of muscle tissue, and increasing adenosine triphosphate (ATP) production [5,6].

**Figure 2:**
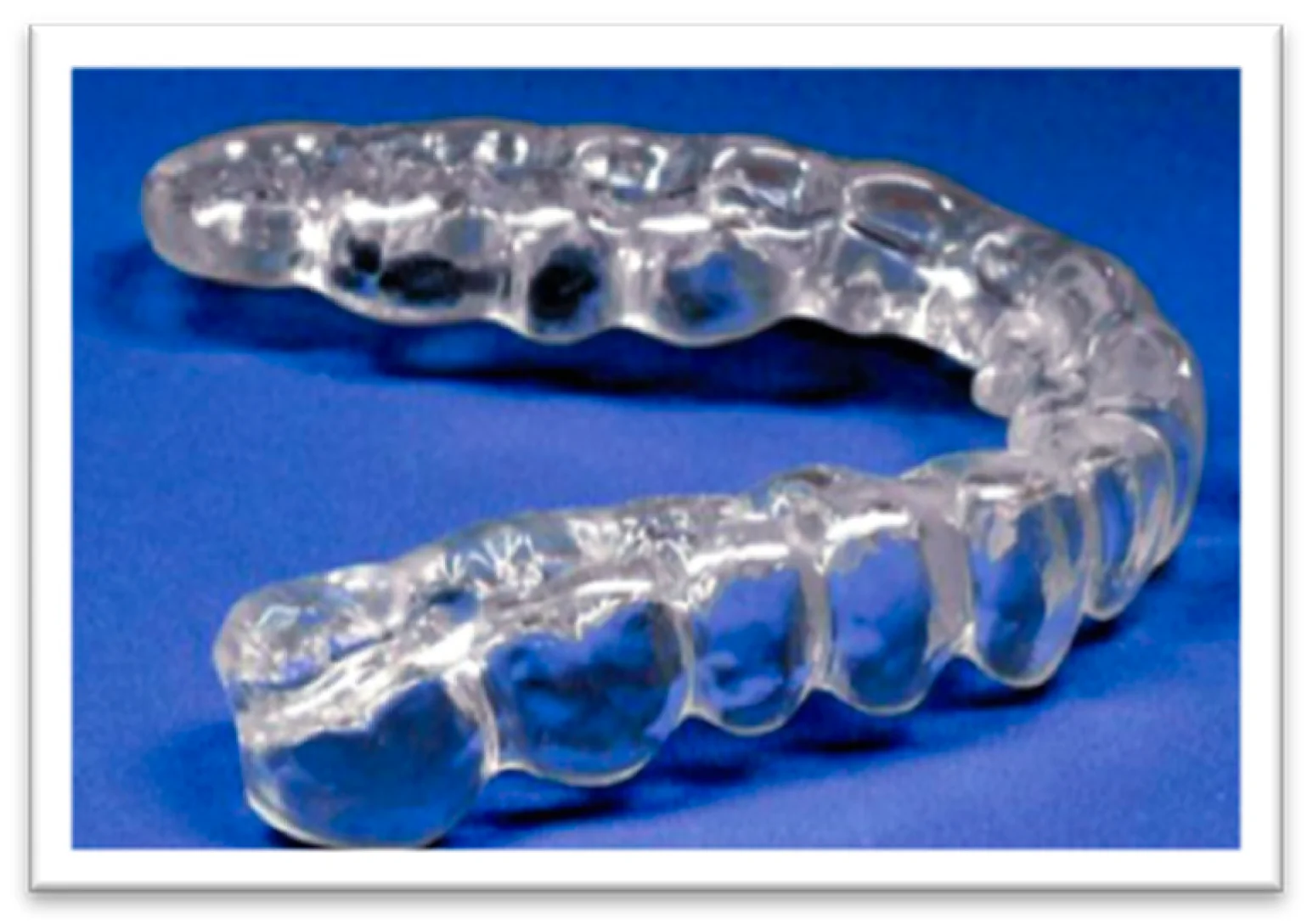
Occlusal splint.

Occlusal splints (bruxoguards) covering the dental arches are commonly used to modify occlusal relationships, redistribute occlusal loading, and prevent excessive tooth wear and mobility. In addition, they reduce bruxism and other parafunctional activities and constitute an established conservative treatment modality for masticatory muscle pain, temporomandibular joint (TMJ) pain, and temporomandibular disorders (Figure 2) [7,8,9].

Research Hypothesis

The working hypothesis was that low-level laser therapy would produce a significant reduction in pain intensity and improvement in quality of life in patients with temporomandibular disorders, with treatment-related changes evaluated relative to those observed following conventional splint-medication therapy.

Study Aim

The primary aim of this study was to evaluate the efficacy of low-level laser therapy in reducing pain intensity among patients diagnosed with temporomandibular disorders.

Specific Objectives

- To compare the effects of low-level laser therapy and conventional splint-medication therapy on pain intensity in patients with temporomandibular disorders.
- To assess changes in patients’ quality of life following treatment and to evaluate the association between pain intensity and quality-of-life outcomes.

## 2. Materials and Methods

### 2.1. Therapeutic Protocols

**Figure 3:**
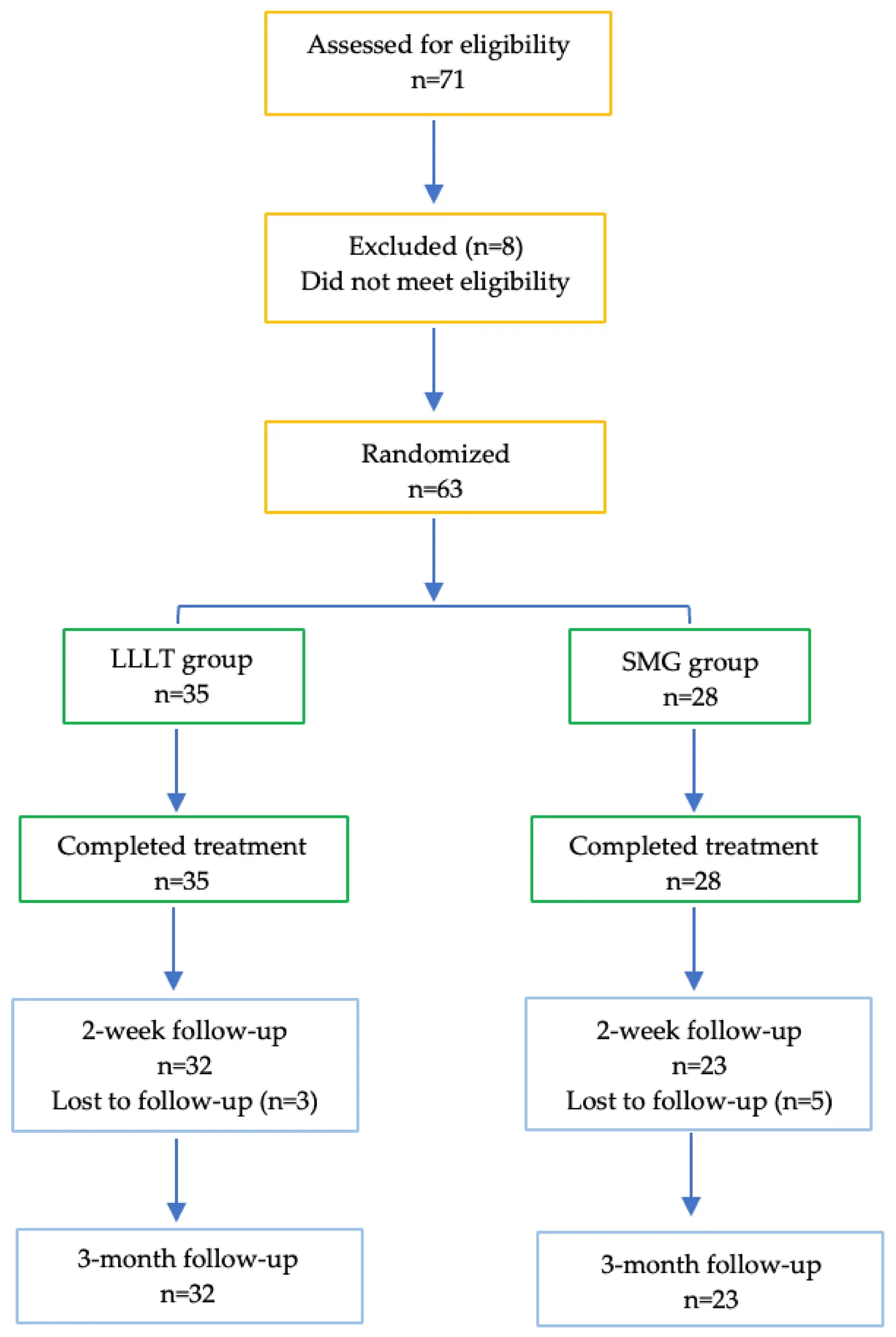
Flow diagram of participant recruitment and follow-up.

This prospective clinical study evaluated the overall improvement in patients with temporomandibular disorders (TMD) following two different therapeutic approaches. A total of 71 consecutive patients with a clinical diagnosis of TMD, established according to the Research Diagnostic Criteria for Temporomandibular Disorders (RDC/TMD) proposed by Dworkin and Le Resche, were assessed for eligibility between 2016 and 2024 at the Department of Prosthodontics, School of Dental Medicine, University of Belgrade. After application of the predefined inclusion and exclusion criteria, 63 eligible participants were enrolled and randomly allocated to the low-level laser therapy group (LLLTG) or the splint-medication group (SMG) (Figure 3). The simple randomization with allocation ratio based on a random number table was used. Written informed consent to participate and publish data was obtained from all participants.

The first group (LLLTG) (35 patients, 55.6%) was treated with low-level laser therapy (LLLT) according to the recommended protocol. The control Splint-Medication Group (SMG) (28 participants, 44.4%) received conventional treatment following a standard protocol, which included premedication with nonsteroidal anti-inflammatory drugs (NSAIDs) to reduce pain and facilitate mandibular opening prior to occlusal splint insertion, followed by occlusal splint therapy. The occlusal splint was used to redistribute occlusal forces, reduce the load on temporomandibular joint (TMJ) structures, and improve mandibular function. Patient demographics indicate similarity of the groups’ Age (Mean ± SD) LLLTG 45.77 ± 18.72, SMG 38.75 ± 14.4, and Sex, *n* (%) Male LLLTG 4 (11.4%) SMG3 (10.7%), Female LLLTG 31 (88.6%), SMG 25 (89.3%).

The inclusion criteria:

- pain in the preauricular region;
- pain or tenderness on palpation of the masticatory muscles;
- pain or tenderness on palpation of the lateral poles of the mandibular condyles;
- limited and painful mandibular movements;
- mandibular stiffness accompanied by pain.

The exclusion criteria:

- patients who had undergone any form of TMD therapy within the previous three months;
- patients with a history of traumatic head and neck injuries or craniofacial anomalies of known or unknown etiology;
- patients in whom clinical examination indicated that the pain was of dentogenic, otogenic, neurogenic, or vascular origin, or associated with neoplastic lesions in adjacent structures (ear, throat, eye, nose, or paranasal sinuses);
- patients with chronic systemic diseases that could compromise their general health status and potentially confound the clinical presentation of TMD;
- female patients who were pregnant or planning pregnancy;
- patients younger than 20 years of age;
- patients who did not provide informed consent to participate in the study.

An a priori sample-size calculation was performed using G*Power software (version 3.1). The calculation was based on the primary outcome and on an anticipated medium-to-large between-group treatment effect derived from previously published randomized clinical trials evaluating low-level laser therapy in patients with temporomandibular disorders [10,11,12,13].

Assuming a two-sided significance level of α = 0.05 and statistical power of 80%, the minimum required sample size was estimated to be approximately 60 participants. A total of 63 participants were enrolled in the study.

### 2.2. Clinical Examination and RDC/TMD Protocol

A detailed clinical examination of the orofacial system was performed in all subjects in order to determine the presence of symptoms and signs of TMD. Patients with symptoms and signs of TMD were included in the study. All subjects were healthy and thoroughly informed of the research protocol. Clinical examination and functional analysis of the orofacial system were based on the diagnostic protocol Research Diagnostic Criteria for TMD RDC/TMD—Supplementary Material S1 [13], one or more symptoms of painful muscle and/or joint dysfunction were recorded:

1. Pain in the Preauricular Region,
2. Pain or Sensitivity When Palpating the Masticatory Muscles,
3. Limited And/or Painful Movements of the Lower Jaw,
4. Deflection of the Lower Jaw During Mouth Opening and
5. Presence of Sound Phenomena When Opening the Mouth.

The clinical examination and assessment of pain were conducted in accordance with the RDC/TMD protocol. The primary pain outcome was the RDC/TMD Axis II characteristic pain intensity score, derived from questions 7–9 of the questionnaire. Oral-health-related quality of life was assessed using the OHIP-14 questionnaire.

#### Laser and Conventional Treatment Protocol

LLLT is non-invasive therapeutic modality with analgesic, anti-inflammatory, and bio-stimulative effects [14,15]. The study group included 35 participants subjected to low-level laser therapy (LLLT), using the following protocol three times a week, for five weeks. Gallium-aluminum-arsenide (GaAlAs) semiconductor diode laser Eco Medico Laser, Electronic Design, Belgrade, Serbia, was used, with a maximum output power of 120 mW and operating output power of 70 mW. Laser parameters were set as follows: wavelength, 780 nm; probe aperture, 1 cm^2^; time, 60 s per point. The power density was 70 mW/cm^2^, radiant energy 4.2 J per point, daily energy delivered 16.8 J, and energy density dose 4.2 J. Dose per session was 16.8 J and cumulative dose 252 J per treatment. The pulse current mode was set to a frequency of 1600 Hz. During the treatment, both the patients and the therapist wore protective glasses with a filter. Application of the laser probe is performed by placing it orthogonally on the skin in the region that was painful on palpation, on a total of four most painful points that were registered by the patient’s clinical examination. Application duration was 4 min per session. Within the LLLT group, evaluations were performed before therapy, after the 5th and 10th therapy session, immediately after the end of therapy, two weeks after therapy, and three months after therapy. A total of 15 visits were performed three times a week over five weeks. The first three visits were scheduled on three consecutive days.

The control group included 28 participants subjected to combined medicament and occlusal splint therapy: nonsteroidal anti-inflammatory drug—NSAID ibuprofen (Brufen, Viatris Healthcare d.o.o., Blegrade, Serbia) for two weeks (first three days 3 × 400 mg, followed by 2 × 400 mg for the remaining period) in order to reduce pain and facilitate occlusal splint therapy, which was prescribed for one month. The medications were used following the manufacturers indications. There were no patients with allergies, systemic diseases, or pregnant patients for potential adverse effects.

The maxillary stabilization splint was made using a three-layer thermoplastic Ercoloc-pro material (Erkodent Erich Kopp GmbH, Pfalzgrafenweiler, Germany) with a thickness of 3 mm. The patient was instructed to wear the splint according to a gradual adaptation protocol. On the first day, the splint was worn for 30 min. The duration of use was then increased by 30 min each subsequent day until the patient achieved a continuous wearing period of 4 h. Following successful adaptation to 4 h of continuous wear, the splint was prescribed for overnight use. After establishing tolerance to nocturnal wear, the splint could additionally be used during daytime.

There were no adverse events during or after treatment. All patients were treated at the School of Dental Medicine’s Clinic for Prosthetics in Belgrade as part of scientific research. Faculty resources were used and patients were not charged for treatment costs.

Pain intensity was assessed using the RDC/TMD characteristic pain intensity score at the predefined evaluation time points [16,17].

### 2.3. Oral Health Related Quality of Life Assessment

Oral health-related quality of life was evaluated using the Oral Health Impact Profile questionnaire (OHIP-14) [18]. Score evaluation was conducted according to the questionnaire scale, at only two time points prior to therapy (i.e., baseline and three months after therapy) with the goal of assessing pain intensity and its influence on quality of life (Supplementary Material S2). The respondents filled out the questionnaire before the therapy and three months after the end of the therapy. The OHIP-14 questionnaire contains 14 questions with offered answers graded on a five-point Likert scale.

Respondents answered the question: “In the last month, how often were the following problems present?” Values from 0 to 4 indicate the following answers:

not at all ………….. 0

rarely……………... 1

occasionally............ 2

often……………..... 3

very often…...…..... 4

The final score on the questionnaire was obtained by adding the marked values for all 14 questions. The minimum score was 0 and the maximum score was 56. A higher score indicated a lower level of quality of life.

The questionnaires were filled out by patients treated by doctors specialized in dental prosthetics who did not participate in the study, and thus had no influence on the results.

### 2.4. Pain Intensity and Psychological Assessment

The primary outcome was pain intensity assessed using the Research Diagnostic Criteria for Temporomandibular Disorders (RDC/TMD) Axis II characteristic pain intensity (CPI) score. Participants rated: (1) their current pain intensity, (2) the worst pain intensity experienced during the previous six months, and (3) the average pain intensity experienced during the previous six months. Each item was rated on an 11-point numerical rating scale ranging from 0 (“no pain”) to 10 (“pain as bad as could be”). The CPI score was calculated as the arithmetic mean of the three item scores multiplied by 10, yielding a total score ranging from 0 to 100. Higher scores indicated greater pain intensity.

Therapeutic outcomes were classified according to the percentage reduction in the RDC/TMD characteristic pain intensity score from baseline to immediately after completion of therapy. A successful therapeutic outcome was defined as a reduction in pain intensity of at least 30% from baseline. Treatment failure was defined as a reduction of <30%, moderate improvement as a reduction of ≥30% to <50%, and substantial improvement as a reduction of ≥50%.

### 2.5. Statistical Analysis

Statistical analysis was performed using IBM SPSS Statistics v22 software (SPSS Inc., Chicago, IL, USA). Descriptive statistics were presented as mean ± standard deviation for normally distributed variables and as median with minimum and maximum values or interquartile ranges (Q1–Q3) for non-normally distributed data. Differences between independent groups were assessed using the Mann–Whitney U test, while longitudinal within-group comparisons were performed using the Friedman test followed by post hoc Wilcoxon signed-rank analysis for pairwise comparisons between evaluation periods.

Longitudinal changes in the RDC/TMD characteristic pain intensity (CPI) score were additionally evaluated using a baseline-adjusted linear mixed-effects model. The model included treatment group, post-treatment assessment time, the treatment group × time interaction, and baseline CPI score as fixed effects, with participant as a random effect. The mixed-effects model incorporated all available longitudinal observations without imputation of missing follow-up data.

Changes in oral health-related quality of life (OHIP-14) were analyzed using a repeated-measures general linear model. Because this analysis requires complete observations at both assessment points, only participants with available OHIP-14 data at baseline and at the three-month follow-up were included (complete-case analysis). The model included treatment group as the between-subject factor, time as the within-subject factor, and the treatment group × time interaction.

A *p* value < 0.05 was considered statistically significant.

This study was approved by the School of Dental Medicine Ethics Committee, University of Belgrade, number 36/33.

## 3. Results

Comparison of RDC/TMD characteristic pain intensity scores between the LLLTG and SMG revealed no significant between-group differences either before treatment (Z = −0.80, *p* = 0.425) or after treatment (Z = −1.39, *p* = 0.166), indicating comparable pain levels at both assessment time points (Table 1).

**Figure 4:**
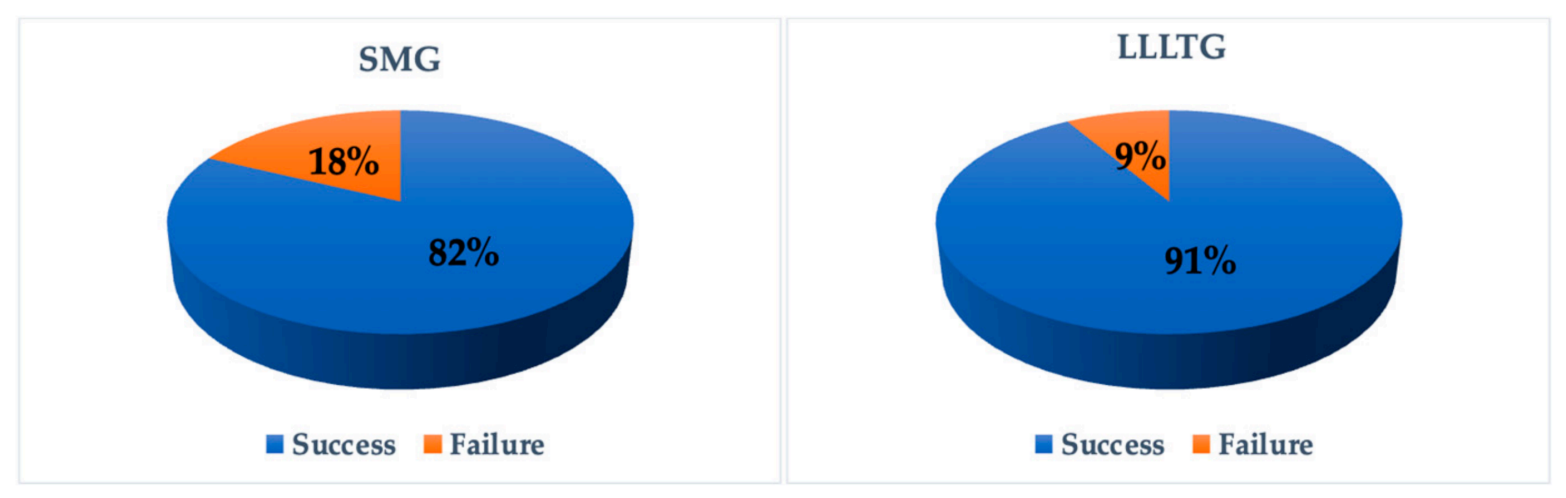
Distribution of therapeutic outcomes based on the percentage reduction in the RDC/TMD characteristic pain intensity score from baseline to immediately after therapy.

As shown in Figure 4, the proportion of patients achieving a successful therapeutic outcome was greater in the LLLTG (91.4%) than in the SMG (82%); however, this difference was not statistically significant.

**Figure 5:**
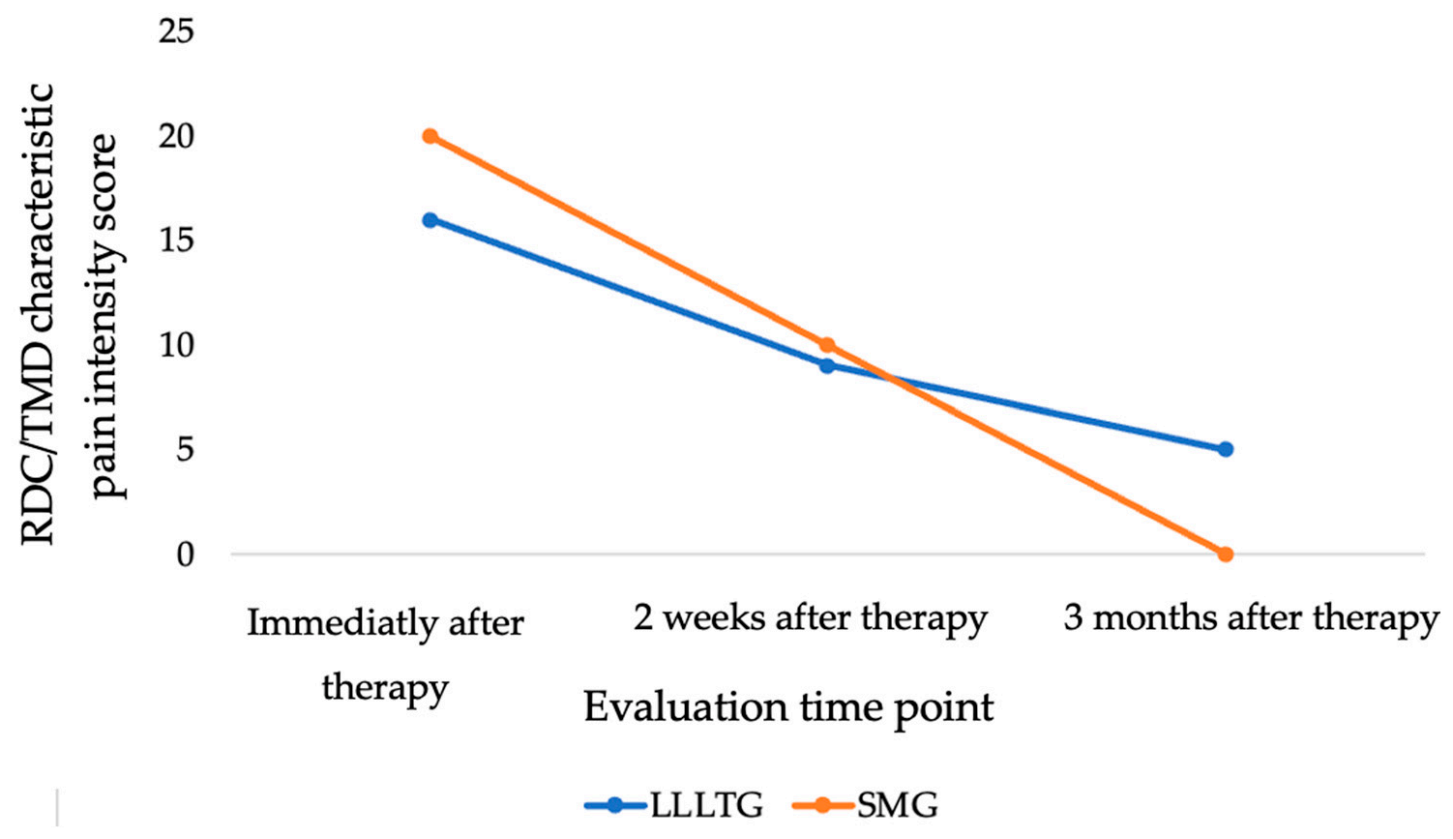
Secondary descriptive analysis of post-treatment RDC/TMD characteristic pain intensity scores in patients with successful therapeutic outcomes.

Figure 5 shows the post-treatment changes in the median RDC/TMD characteristic pain intensity score among patients with successful therapeutic outcomes. This is a secondary descriptive analysis and is presented to illustrate pain changes during the follow-up period in this subgroup.

A statistically significant reduction in RDC/TMD characteristic pain intensity score over time was observed in both treatment groups (LLLTG and SMG) (*p* < 0.01) (Table 2). Descriptive analyses at T4 and T5 were based on participants who attended the scheduled follow-up visits. That is why subsequent assessment was conducted on a reduced sample. Owing to the characteristics of the treatment protocols, intermediate assessments at T1 and T2 were performed exclusively in the LLLTG.

The overall treatment × time interaction reached statistical significance, indicating that the longitudinal trajectories were not completely identical. However, baseline-adjusted comparisons at the individual post-treatment assessment points did not demonstrate statistically significant between-group differences (Table 3).

After adjustment for multiple comparisons using the Holm procedure, all significant within-group reductions in RDC/TMD characteristic pain intensity scores in the LLLTG remained statistically significant (Table 4). Similarly, Holm-adjusted post hoc analyses confirmed that the significant reductions in RDC/TMD characteristic pain intensity scores observed in the SMG remained statistically significant after correction for multiple testing (Table 5).

Because the linear mixed-effects model addressed the primary longitudinal comparison between the treatment groups, the Friedman and Wilcoxon analyses were performed as complementary within-group analyses to describe changes over time within each treatment group.

In line with that, post hoc Wilcoxon signed-rank analysis demonstrated statistically significant reductions in RDC/TMD characteristic pain intensity score between baseline and all subsequent evaluation time points in the LLLT group: after the 5th session (Z = −4.71, *p* < 0.01), after the 10th session (Z = −5.01, *p* < 0.01), immediately after therapy (Z = −5.09, *p* < 0.01), two weeks after therapy (Z = −4.94, *p* < 0.01), and three months after therapy (Z = −4.94, *p* < 0.01). Significant reductions were also observed between the 5th and 10th treatment sessions (Z = −2.75, *p* < 0.01), between the 5th session and immediately after therapy (Z =−4.12, *p* < 0.01), two weeks after therapy (Z = −4.02, *p* < 0.01), and three months after therapy (Z =−4.52, *p* < 0.01). Furthermore, significant reductions were found between the 10th session and immediately after therapy (Z = −3.37, *p* < 0.01), two weeks after therapy (Z =−3.81, *p* < 0.01), and three months after therapy (Z =−4.32, *p* < 0.01). No significant differences were observed between measurements obtained immediately after therapy and two weeks after therapy (Z = −1.52, *p* = 0.129), or between the two-week and three-month follow-up evaluations (Z = −1.77, *p* = 0.076). However, a statistically significant reduction was observed between measurements obtained immediately after therapy and three months after therapy (Z = −2.56, *p* = 0.01) (Table 6).

**Figure 6:**
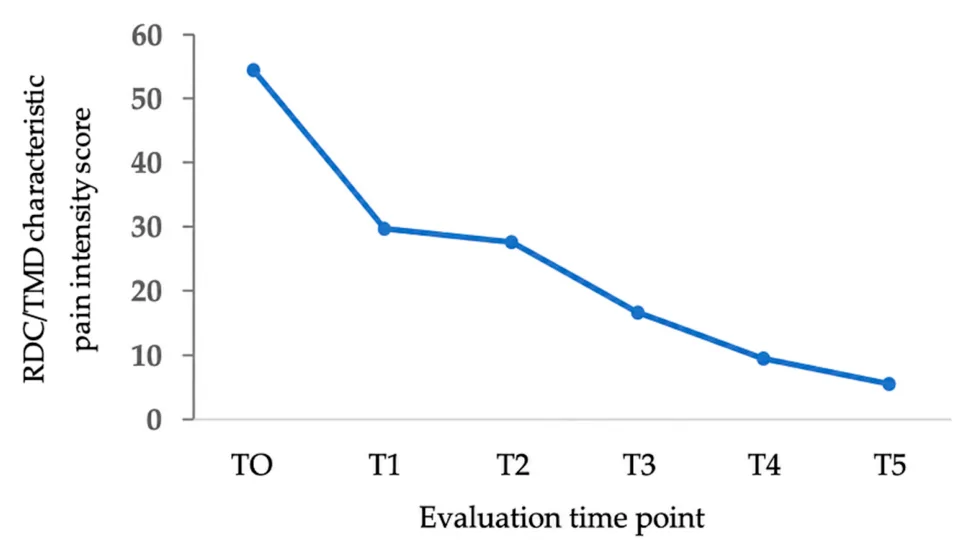
RDC/TMD Characteristic Pain Intensity score in the LLLTG at different evaluation periods.

Figure 6 illustrates a progressive reduction in RDC/TMD characteristic pain intensity score in the LLLTG across all evaluation time points, with the lowest pain intensity observed three months after completion of therapy.

Similar to the findings observed in the LLLTG, post hoc Wilcoxon signed-rank analysis in the SMG demonstrated statistically significant reductions in RDC/TMD characteristic pain intensity score between baseline and all follow-up evaluations (*p* < 0.01). However, no statistically significant difference was observed between the two-week and three-month follow-up evaluations (*p* = 0.157) (Table 7).

**Figure 7:**
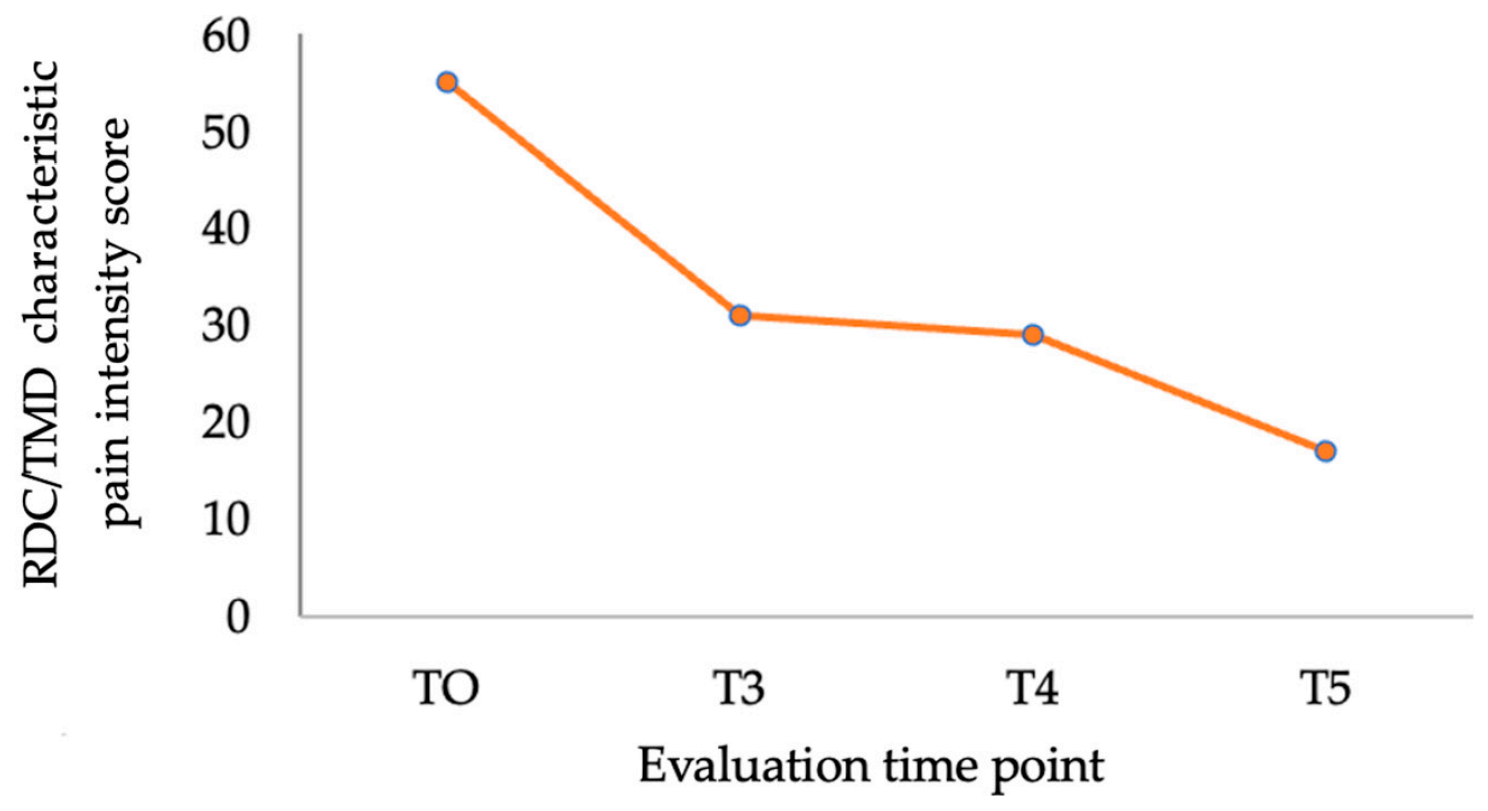
RDC/TMD characteristic pain intensity score in the SMG group at different evaluation periods.

Figure 7 demonstrates a progressive reduction in RDC/TMD characteristic pain intensity score in the SMG group across all evaluation time points, with the lowest pain intensity recorded three months after therapy.

There was no statistically significant association between pain duration and treatment outcome (*p* = 0.850).

Table 8 summarizes the distribution of treatment outcomes according to the duration of pain symptoms.

Table 9 presents the descriptive parameters of OHIP-14 scores before and after therapy in the LLLTG and SMG. A substantial reduction in median OHIP-14 scores was observed after therapy in both groups, indicating improved oral health-related quality of life following treatment.

Table 10 shows the comparison of OHIP-14 scores before and after therapy in patients treated with LLLTG and conventional therapy (SMG) using a repeated-measures general linear model. A statistically significant reduction in OHIP-14 scores was observed after therapy compared with baseline (F = 172.17, *p* < 0.01), indicating a significant improvement in oral health-related quality of life following treatment. No statistically significant differences were observed between the two treatment groups (F = 0.09, *p* = 0.925), and no significant interaction between treatment group and time was detected (F = 0.04, *p* = 0.842).

**Figure 8:**
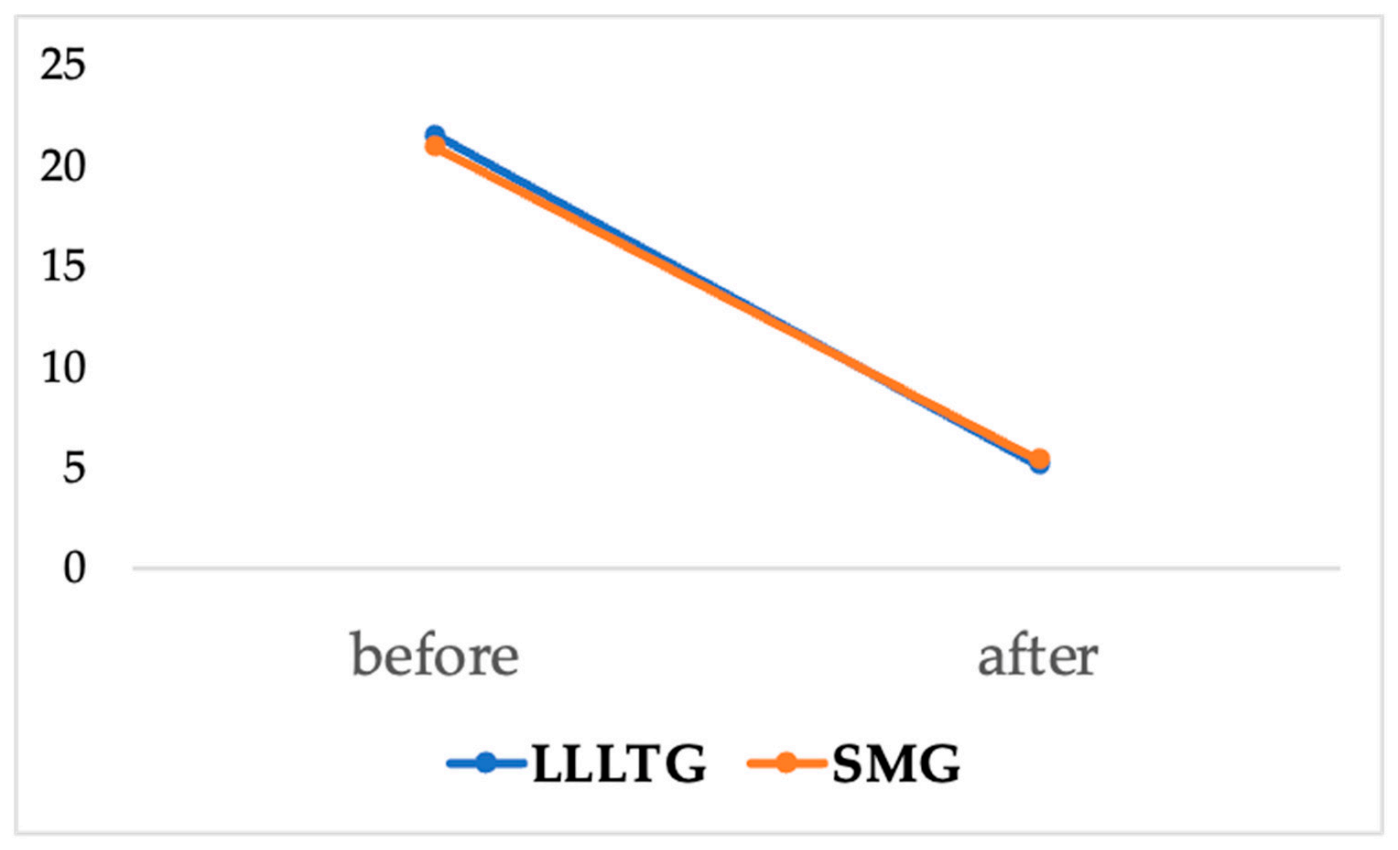
Mean OHIP-14 scores before and after therapy in patients treated with LLLTG and SMG.

Figure 8 demonstrates a marked decrease in OHIP-14 scores after therapy compared with baseline. The curves for the two treatment groups almost completely overlap, indicating no significant differences between the groups according to the type of therapy.

Table 11 presents the correlation between RDC/TMD characteristic pain intensity score and OHIP-14 scores before and after therapy. A significant positive correlation was observed in the overall sample both before therapy (r = 0.33, *p* = 0.008) and three months after therapy (r = 0.47, *p* < 0.01), indicating that higher pain intensity was associated with poorer oral health-related quality of life. At baseline, the correlation was significant only in the SMG, whereas at the three-month follow-up it remained significant only in the LLLTG.

## 4. Discussion

Temporomandibular disorders (TMDs) comprise a heterogeneous group of conditions affecting the temporomandibular joint (TMJ), masticatory muscles, and associated soft and hard tissues, including tendons, nerves, and bones. Pain is the predominant clinical symptom and is frequently accompanied by functional impairment, resulting in a reduced oral health-related quality of life. Despite extensive research, the diagnosis and management of TMD remain challenging because of its multifactorial etiology and highly variable clinical presentation. While some signs and symptoms resolve spontaneously, others persist for years despite appropriate treatment. Consequently, numerous therapeutic modalities have been proposed, ranging from conservative to invasive approaches. Although many of these interventions provide symptomatic relief, their long-term effectiveness remains uncertain, and no single treatment has been shown to be universally effective for the diverse clinical manifestations of TMD.

The contemporary management of TMD involves a multimodal approach in which different therapeutic modalities are applied either simultaneously or sequentially, depending on the patient’s clinical presentation. Treatment aims not only to reduce pain and inflammation within the temporomandibular joint and associated musculature, thereby improving function and preventing disease progression, but also to address the underlying etiological factors whenever possible. A wide range of conservative treatment options is available, including pharmacological therapy with muscle relaxants and nonsteroidal anti-inflammatory drugs (NSAIDs), occlusal splint therapy, platelet-rich plasma injections, neuromodulator therapy, therapeutic exercises, ultrasound therapy, behavioral interventions, low-level laser therapy (LLLT), and light-emitting diode (LED) therapy. Irreversible interventions, such as occlusal equilibration by selective grinding and surgical procedures, are generally reserved for carefully selected patients [19,20,21].

In the present prospective study, conventional splint-medication therapy, consisting of NSAID premedication followed by occlusal splint therapy, was selected as the reference treatment because of its well-established efficacy in the management of painful TMD. Its therapeutic outcomes were compared with those of low-level laser therapy to determine whether LLLT was associated with significant pain reduction and improvement in oral health-related quality of life. No statistically significant baseline-adjusted between-group differences were observed at the individual post-treatment assessment points. Although the longitudinal response patterns were not completely identical between the treatment groups, the absence of statistically significant baseline-adjusted between-group differences at the individual follow-up assessments should not be interpreted as evidence of therapeutic equivalence, as the present study was not designed as an equivalence or non-inferiority trial. Instead, the results indicate that both treatment modalities were associated with significant clinical improvement despite their different longitudinal response patterns.

Low-level laser therapy (LLLT) has been widely used in the management of various musculoskeletal disorders, including osteoarthritis, tendinitis, epicondylitis, myofascial pain, acute and chronic neck pain, and fibromyalgia. Owing to the similarities in the pathophysiological mechanisms underlying pain and inflammation in musculoskeletal tissues, LLLT has also been applied in the treatment of temporomandibular disorders. Its proposed mechanisms include photo-bio-modulation-induced anti-inflammatory, analgesic, and bio-stimulatory effects, which contribute to pain reduction and improved function of the temporomandibular joint and masticatory muscles. These findings are consistent with previously published studies demonstrating the clinical efficacy of LLLT in patients with TMD [14,15,22,23].

In our research in intergroup assessment no significant differences in average pain intensity between the two groups were found before the therapy, which indicates the homogeneity of the sample and that there were no significant differences in the severity of pain in the two groups. Furthermore, there were no significant differences in pain intensity in the two assessed groups after therapy, which suggests that the therapeutic effect of different therapeutic modalities in both groups was similarly successful (Table 1).

The findings of the present study are consistent with those reported by [7], in which the most common reasons for consultation were masticatory muscle disorders, particularly involving the masseter and lateral pterygoid muscles, and limitation of mouth opening. Treatment consisted of a combination of muscle relaxation, occlusal equilibration, kinesitherapy, pharmacological therapy, and swallowing re-education. The primary symptom was successfully relieved in 82.3% of patients, although recurrence occurred in 15.7%, highlighting the complex and often chronic nature of TMD. Most patients required four to seven different therapeutic modalities, resulting in prolonged treatment and increased healthcare costs. Similarly, our study demonstrated high therapeutic success rates in both groups (91.4% in the LLLTG and 82.1% in the SMG), with sustained symptom remission throughout the follow-up period. These findings further support the effectiveness of conservative treatment approaches in the management of TMD.

High therapeutic success was achieved with both treatment modalities, with clinically successful outcomes observed in 91.4% of patients treated with LLLT and 82.1% of those receiving conventional splint-medication therapy. Although the success rate was numerically higher in the LLLTG, the difference between the groups was not statistically significant. These findings are consistent with previous studies demonstrating beneficial clinical effects of LLLT in the management of TMD [6,14]. Furthermore, Chellappa and Thirupathy [5] demonstrated significantly greater pain reduction following LLLT than after transcutaneous electrical nerve stimulation (TENS) (*p* < 0.01), further supporting the analgesic potential of photobiomodulation in patients with TMD.

Pain intensity decreased progressively throughout the course of LLLT, with a statistically significant reduction observed across all evaluation time points compared with baseline (*p* < 0.01). These findings indicate that the analgesic effect of LLLT developed gradually during treatment and was maintained throughout the follow-up period, suggesting a sustained therapeutic benefit. The progressive reduction in pain is consistent with the proposed biological effects of photobiomodulation, including modulation of inflammatory mediators, enhanced tissue repair, and improved microcirculation, all of which contribute to pain relief and functional recovery in patients with TMD [14,15,21,23].

The present findings demonstrate that conventional splint-medication therapy produced a sustained reduction in pain intensity throughout the treatment and follow-up periods. Most of the therapeutic improvement occurred during active treatment and within the first two weeks after its completion, after which pain levels remained stable, indicating maintenance of the analgesic effect. These findings support the effectiveness of conventional conservative therapy in achieving durable pain control in patients with TMD. Similar outcomes have been reported in previous studies, which have shown that splint therapy combined with pharmacological treatment provides effective short-term pain relief and functional improvement, although its long-term superiority over other conservative modalities remains uncertain [24,25,26].

An additional finding of the present study was that the duration of pain symptoms did not significantly influence treatment outcome.

Patients with symptoms lasting longer than six months achieved similar outcomes to those with a shorter duration of symptoms, suggesting that symptom chronicity did not significantly influence treatment outcomes following either LLLT or conventional splint-medication therapy. This observation is clinically relevant because it indicates that conservative treatment should not be withheld solely on the basis of prolonged symptom duration. Nevertheless, larger prospective studies with longer follow-up periods are required to confirm these findings and to determine whether symptom duration influences long-term treatment outcomes.

The present findings indicate that the duration of pain symptoms did not significantly influence treatment success. Although patients with symptoms lasting less than six months exhibited a slightly higher therapeutic success rate than those with longer symptom duration, the difference was not statistically significant. These results suggest that both LLLT and conventional splint-medication therapy may be effective irrespective of symptom chronicity. Clinically, this finding supports the use of conservative treatment even in patients with longstanding TMD symptoms, although larger studies are warranted to confirm these observations.

Both treatment modalities were associated with a significant improvement in oral health-related quality of life, as reflected by the marked reduction in OHIP-14 scores after therapy. This finding suggests that effective pain control and functional recovery positively influenced patients’ overall well-being.

The significant improvement in oral health-related quality of life observed after treatment was accompanied by a reduction in pain intensity, reinforcing the positive correlation between pain severity and OHIP-14 scores. These findings indicate that effective pain control is closely associated with improved daily functioning and enhanced oral health-related quality of life.

Numerous treatment modalities have been proposed for the management of TMD, reflecting the multifactorial nature of the disorder. A recent systematic review and network meta-analysis [27] compared the effectiveness of available interventions with respect to pain reduction and improvement in maximum mouth opening. The authors concluded that minimally invasive procedures, particularly when combined with adjunctive pharmacological therapy, were more effective than conservative treatments in achieving short- and intermediate-term improvements in pain and mandibular function. Based on these findings, the authors suggested that minimally invasive procedures should be considered early in the treatment algorithm for patients who fail to respond adequately to initial conservative management.

Our previous studies [28,29] demonstrated that persistent TMD symptoms are associated with poorer oral health-related quality of life, higher levels of depression, and reduced work ability. These findings highlight the importance of early diagnosis and individualized therapeutic approaches to prevent symptom progression and mitigate the psychosocial impact of temporomandibular disorders.

Temporomandibular disorders comprise a heterogeneous group of conditions that share several clinical characteristics with other chronic pain disorders. Their etiology is multifactorial, involving complex interactions between biological, biomechanical, psychological, and social factors, making diagnosis and management particularly challenging. While early treatment strategies focused primarily on structural and biomechanical abnormalities, contemporary evidence recognizes the equally important contribution of psychosocial factors to symptom persistence and treatment outcomes. Consequently, a multimodal, multidisciplinary approach that combines patient education, behavioral interventions, physical therapy, pharmacological treatment, and other non-invasive modalities is increasingly recommended as the first-line management strategy. Although most patients respond well to conservative therapy, prolonged continuation of ineffective treatment is not advisable, and minimally invasive surgical procedures should be considered when adequate clinical improvement cannot be achieved [18].

The findings of the present study support this contemporary treatment concept. Both low-level laser therapy and conventional splint-medication therapy were associated with significant reductions in pain intensity and improvements in oral health-related quality of life. However, no statistically significant between-group differences were demonstrated at the individual assessment points. These results emphasize that treatment should be individualized according to each patient’s clinical presentation, with conservative therapies used alone or in combination to maximize their synergistic effects. Such an approach may optimize symptom control, improve functional outcomes, and ultimately enhance patients’ quality of life.

This supports the working hypothesis of this study that low-level laser therapy (LLLT) would produce a significant reduction in pain intensity and improvement in quality of life in patients with temporomandibular disorders, with treatment-related changes evaluated relative to those observed following conventional splint-medication therapy.

The present study has several limitations. Although the initial sample size met the a priori calculated requirement, a small proportion of participants was lost during follow-up, resulting in a reduced number of observations at the later assessment time points. This may have reduced the precision of the long-term estimates. However, to minimize the potential impact of missing follow-up data, a baseline-adjusted linear mixed-effects analysis using all available longitudinal observations was additionally performed. Future studies are warranted to confirm these findings.

## 5. Conclusions

The applied low-level laser therapy protocol was associated with a significant reduction in pain intensity and improvement in oral health-related quality of life in patients with temporomandibular disorders. Although no statistically significant differences between the treatment groups (low-laser therapy and splint-medication group) were demonstrated at the individual post-treatment assessment points, these findings support LLLT as a potentially useful non-invasive conservative treatment option.

## Figures and Tables

**Table 1:** Descriptive statistics of RDC/TMD characteristic pain intensity scores before and immediately after therapy.

	CPI Score (0–100)	Median	Min	Max	*p*
Before therapy	LLLTG	55	30	89	0.425
	SMG	63	30	100	
After therapy	LLLTG	20	0	65	0.166
	SMG	23	0	70	

**Table 2:** Longitudinal evaluation of RDC/TMD characteristic pain intensity score within LLLTG and SMG.

Group	Evaluation Period	Number of Patients	Median	Min	Max	*p*
	T0	35	55	30	89	<0.01
	T1	35	40	10	80	
LLLTG	T2	35	30	0	56	
	T3	35	20	0	65	
	T4	32 **	9	0	60	
	T5	32 **	5	0	50	
SMG	T0	28	63	30	100	
	T3	28	23	0	70	<0.01
	T4	23 **	10	0	40	
	T5	23 **	0	0	35	

Friedman test; ** the number of participants at T4 and T5 reflects those who completed the scheduled follow-up assessments. Participants who did not attend these follow-up visits were excluded from these time-point analyses because of missing follow-up data. T0—before therapy; T1—after the 5th LLLTG session; T2—after the 10th LLLTG session; T3—immediately after therapy; T4—2 weeks after therapy; T5—3 months after therapy.

**Table 3:** Baseline-adjusted longitudinal comparison of RDC/TMD characteristic pain intensity score between treatment groups.

Model Effect/Contrast	Estimate, β	95% CI	*p* Value
Baseline RDC/TMD characteristic pain intensity score	0.40	0.19 to 0.60	<0.001
Time: 2 weeks vs. immediately after therapy	−11.70	−16.46 to −6.94	<0.001
Time: 3 months vs. immediately after therapy	−13.96	−18.72 to −9.20	<0.001
LLLGT vs. SMG immediately after therapy	−5.55	−13.60 to 2.50	0.177
LLLGT vs. SMG at 2 weeks	3.30	−5.05 to 11.60	0.439
LLLGT vs. SMG at 3 months	1.84	−6.51 to 10.19	0.666
Overall treatment × time interaction	Wald χ^2^ = 9.02, df = 2	/	0.011

**Table 4:** Post hoc pairwise comparisons within the LLLT group (Holm-adjusted *p*-values).

Comparison	Original *p*	Holm-Adjusted *p*
Baseline vs. 5th session	2.47 × 10^−6^	2.47 × 10^−6^
Baseline vs. 10th session	5.35 × 10^−7^	2.14 × 10^−6^
Baseline vs. end of the therapy	3.58 × 10^−7^	1.79 × 10^−6^
Baseline vs. 2 weeks	7.58 × 10^−7^	2.34 × 10^−6^
Baseline vs. 3 months	7.80 × 10^−7^	2.34 × 10^−6^

**Table 5:** Post hoc pairwise comparisons within the SMG group (Holm-adjusted *p*-values).

Comparison	Original *p*	Holm-Adjusted *p*
Baseline vs. end of the therapy	5.09 × 10^−6^	1.53 × 10^−5^
Baseline vs. 2 weeks	2.67 × 10^−5^	5.33 × 10^−5^
Baseline vs. 3 months	2.66 × 10^−5^	5.33 × 10^−5^

**Table 6:** Post hoc pairwise comparison of RDC/TMD characteristic pain intensity score in the LLLTG.

Therapy	T0–T1	T0–T2	T0–T3	T0–T4	T0–T5	T1–T2	T2–T3	T3–T4	T3–T5	T4–T5
	*p*	*p*	*p*	*p*	*p*	*p*	*p*	*p*	*p*	*p*
LLLTG	<0.01	<0.01	<0.01	<0.01	<0.01	0.013	<0.01	0.129	0.01	0.076

T0—before the start of therapy, T1—after the 5th visit of LLLTG, T2—after the 10th visit of LLLTG, T3—immediately after therapy, T4—two weeks after therapy, T5—three months after therapy.

**Table 7:** Post hoc pairwise comparison of RDC/TMD characteristic pain intensity score in the SMG.

Therapy	T0–T3	T0–T4	T0–T5	T3–T4	T3–T5	T4–T5
	*p*	*p*	*p*	*p*	*p*	*p*
SMG	<0.01	<0.01	<0.01	<0.01	<0.01	0.157

T0—before the start of the therapy, T3—immediately after therapy, T4—two weeks after therapy, T5—three months after therapy.

**Table 8:** Distribution of treatment outcomes according to pain duration.

	Treatment Outcome		Total	*p*
	Success	Failure		
Pain Duration	*n* (%)	*n* (%)	*n* (%)	
<6 months	26 (89.7)	3 (10.3)	29 (100%)	0.850
>6 months	29 (85.3)	5 (14.7)	34 (100%)	
Overall	55 (87.3%)	8 (12.7%)	63 (100%)	

Fisher exact probability test.

**Table 9:** Descriptive parameters of OHIP-14 scores before and after therapy.

Quality of Life	*n*	Median	Min	Max
**before therapy**				
LLLTG	35	22.00	6	39
SMG	28	19.5	4	44
**after therapy**				
LLLTG	32	3.00	0	17
SMG	23	3.00	0	28

**Table 10:** Results of comparing the total score for quality of life in groups of patients with TMD before and after applied therapy (LLLTG/SMG) using general linear models for repeated measurements.

		*n*	Mean± SD	Within Subjects	F	*p*
Quality of life before therapy	LLLTG	32	20.74 ± 9.14	Time (baseline post-treatment)	172.17	<0.01
	SMG	23	20.32 ± 11.40	Time x therapy	0.04	0.842
	overall	55	20.57 ± 10.04			
Quality of life after therapy	LLLTG	32	5.32 ± 5.50	Between-subjects		
	SMG	23	5.36 ± 6.91	Therapy	0.09	0.925
	overall	55	5.34 ± 6.06			

**Table 11:** Correlation of pain and scores for quality of life in patients before and after therapy.

RDC/TMD Characteristic Pain Intensity Score Before Therapy	*n*	Quality of Life Before Therapy	*p*
overall	63	0.33	0.008 **
group			
LLLT	35	0.22	0.215
SMG	28	0.44	0.02 *
**RDC/TMD characteristic pain intensity score 3 months after therapy**	***n***	**Quality of life after therapy**	***p***
overall	55	0.47	<0.01 **
group			
LLLTG	32	0.48	<0.01 **
SMG	23	0.37	0.089

* <0.05, ** <0.01.

## Data Availability

The original contributions presented in the study are included in the article/Supplementary Material. Further inquiries can be directed to the corresponding author.

## Supplementary Materials

The following supporting information can be downloaded at: https://www.mdpi.com/article/10.3390/life16081305/s1.

## References

[1] Von Korff M., Ormel J., Keefe F.J., Dworkin S.F. (1992). Grading the severity of chronic pain. Pain.

[2] Ismail F., Eisenburger M., Lange K., Schneller T., Schwabe L., Strempel J., Stiesch M. (2016). Identification of psychological comorbidity in TMD-patients. Cranio®.

[3] List T., Jensen R.H. (2017). Temporomandibular disorders: Old ideas and new concepts. Cephalalgia.

[4] Vaddamanu S.K., Alshadidi A.A.F., Aldosari L.I.N., Khalid I., Binduhayyim R.I.H., Shah S.J., Narapureddy B.R. (2026). Integrating multimodal behavioral therapy into temporomandibular disorders management: A randomized attention-controlled trial using DC/TMD axis II outcomes. Behav. Res. Ther..

[5] Chellappa D., Thirupathy M. (2020). Comparative efficacy of low-Level laser and TENS in the symptomatic relief of temporomandibular joint disorders: A randomized clinical trial. Indian J. Dent. Res..

[6] Shousha T., Alayat M., Moustafa I. (2021). Effects of low-level laser therapy versus soft occlusive splints on mouth opening and surface electromyography in females with temporomandibular dysfunction: A randomized-controlled study. PLoS ONE.

[7] Buduru S., Almăşan O., Condor D., Tăut M., Mesaroș A., Manziuc M., Kui A. (2024). Therapeutic challenges in temporomandibular disorders. Med. Pharm. Rep..

[8] Shenoy M., Shivakumar G.C., Gupta P., Abdul N.S., Prakash J., Ranvijay K., Devi L.S. (2022). Assessment of Symptoms Associated with Temporomandibular Dysfunction and Bruxism among Elderly Population: An Epidemiological Survey. J. Contemp. Dent. Pr..

[9] Poorna T.A., John B., Joshna E.K., Rao A. (2022). Comparison of the effectiveness of soft and hard splints in the symptomatic management of temporomandibular joint disorders: A randomized control study. Int. J. Rheum. Dis..

[10] Ahrari F., Madani A.S., Ghafouri Z.S., Tunér J. (2014). The efficacy of low-level laser therapy for the treatment of myogenous temporomandibular joint disorder. Lasers Med. Sci..

[11] Madani A., Ahrari F., Fallahrastegar A., Daghestani N. (2020). A randomized clinical trial comparing the efficacy of low-level laser therapy (LLLT) and laser acupuncture therapy (LAT) in patients with temporomandibular disorders. Lasers Med. Sci..

[12] Chamani G., Zarei M.R., Rad M., Mafi S. (2024). Comparison of low-level laser therapy and standard treatment for temporomandibular disorders: An assessment of therapeutic and placebo effects. J. Oral Rehabil..

[13] Anderson G.C., Gonzalez Y.M., Ohrbach R., Truelove E.L., Sommers E., Look J.O., Schiffman E.L. (2010). The Research Diagnostic Criteria for Temporomandibular Disorders. VI: Future directions. J. Orofac. Pain..

[14] Ansari S., Charantimath S., Lagali-Jirge V., Keluskar V. (2024). Comparative efficacy of low-level laser therapy (LLLT) to TENS and therapeutic ultrasound in management of TMDs: A systematic review & meta-analysis. Cranio®.

[15] Bhoi S., Koushal D., Manas A., Sidhu R., Shergill N.K., Kakkad A., Misra K.K., Gupta A.K. (2025). Efficacy of TENS, ultrasound and low level laser in the management of TMJ disorder. Bioinformation.

[16] Reis P.H.F., Laxe L.A.C., Lacerda-Santos R., Münchow E.A. (2022). Distribution of anxiety and depression among different subtypes of temporomandibular disorder: A systematic review and meta-analysis. J. Oral Rehabil..

[17] Shrivastava M., Battaglino R., Ye L. (2021). A comprehensive review on biomarkers associated with painful temporomandibular disorders. Int. J. Oral Sci..

[18] Kovačević I., Barać I., Poljak K.M., Čandrlić S., Čandrlić M. (2025). Assessing the Oral-Health-Related Quality of Life in Patients with Dental Prosthetics: A Cross-Sectional Study from Eastern Croatia. Oral.

[19] Zhang C., Shi K., Zheng Y. (2025). Methodological Considerations for TMD Research: A Call for More Robust Assessment and Analysis. J. Pain Res..

[20] Tian Y., Tan Y., Yang M., Lv X., Zheng Y., Zhang Q., Sun Y., Wang J., Xiong X. (2024). The association between specific oral behaviors and the number of temporomandibular disorder symptoms in the general population: A cross-sectional study. J. Pain Res..

[21] Li D.T.S., Leung Y.Y. (2021). Temporomandibular disorders: Current concepts and controversies in diagnosis and management. Diagnostics.

[22] Argueta-Figueroa L., Flores-Mejía L.A., Ávila-Curiel B.X., Flores-Ferreyra B.I., Torres-Rosas R. (2022). Nonpharmacological Interventions for Pain in Patients with Temporomandibular Joint Disorders: A Systematic Review. Eur. J. Dent..

[23] Ferrillo M., Ammendolia A., Paduano S., Calafiore D., Marotta N., Migliario M., Fortunato L., Giudice A., Michelotti A., de Sire A. (2022). Efficacy of rehabilitation on reducing pain in muscle-related temporomandibular disorders: A systematic review and meta-analysis of randomized controlled trials. J. Back Musculoskelet. Rehabil..

[24] Mahammadli E., Yilmaz O., Girgin F., Ulubay E. (2026). Effects of Low-Level Laser Therapy at Different Energy Densities in Patients with Temporomandibular Disorders: A Randomized Clinical Trial. Appl. Sci..

[25] Miletic A., Lazic Z., Todorovic A., Djordjevic I., Popovic D., Lazic V. (2017). Stress assessment in patients with clinically diagnosed sleep bruxism. Vojn. Pregl..

[26] Beaumont S., Garg K., Gokhale A., Heaphy N. (2020). Temporomandibular Disorder: A practical guide for dental practitioners in diagnosis and management. Aust. Dent. J..

[27] Al-Moraissi E.A., Wolford L.M., Ellis E., Neff A. (2020). The hierarchy of different treatments for arthrogenous temporomandibular disorders: A network meta-analysis of randomized clinical trials. J. Cranio-Maxillofac. Surg..

[28] Djordjevic I., Ivanjac F., Popovic-Antic D., Milicic-Lazic M., Colic M., Zupac L. (2026). Work ability impairment in patients with temporomandibular dysfunction. Srp. Arh. Celok. Lek..

[29] Djordjevic I., Ivanjac F., Popovic-Antic D., Milicic-Lazic M., Colic M., Zupac L. (2026). Association between depression and oro-facial pain: A pilot study. Srp. Arh. Celok. Lek..

