# Supplementary material for: Therapeutic Effect of Low-Level Laser Therapy in Patients with Temporomandibular Disorder
Source: Life (Basel). 2026 Aug 9;16(8):1305. doi: 10.3390/life16081305 (PMC13514900; doi:10.3390/life16081305)
Supplement: Supplementary file 1 [file life-16-01305-s001.zip › life-4437679-supplementary.pdf]

Supplementary 1.

RDC/TMD protocol diagnostic criteria for the investigation of temporomandibular disorders - RDC/TMD (The Research Diagnostic Criteria for Temporomandibular Disorders)

DATE................................OF INSTITUTION....................PATIENT NO..NAMES SURNAME.. SEX............................... YEAR OF BIRTH................OCCUPATION...TEL.............

AXIS II

READ EACH QUESTION CAREFULLY AND CIRCLE ONLY ONE ANSWER

1. How would you rate your general state of health: excellent, very good, good, satisfactory, or bad? excellent.....................1 very good...................2 good........................3 satisfactory........4 bad..................................5

2. How would you rate the condition of your oral cavity: excellent, very good, good, satisfactory, or bad? excellent...................1 very good..................2 good.......................3 satisfactory.........4 bad...........................5

3. In the last 6 months, have you felt pain in areas of the face, jaw, temple, in front of the ear, or in the ear itself? No...................0 Yes...................1 (if you have not felt pain in the last 6 months, go to question no. 14)

4a. How many years ago did you first feel such pain? .... years (if the pain first occurred less than a year ago, skip the previous question and answer the following)

4b. How many months ago did you first feel such pain? ....months

5. Is the pain constant, occasional, or has it appeared only once? Constant..................................1 Occasional...........................2 Only appeared once.....3

6. Have you ever sought medical help for this? No..1 Yes, in the past 6 months..................2 Yes, more than 6 months ago..................3

7. How would you rate your current pain on a scale of 0–10, where the value 0 corresponds to a state without pain, and the value 10 to a state of unbearable pain? (no pain) (excruciating pain) 0 1 2 3 4 5 6 7 8 9 10

8. In the last 6 months, at what intensity on a scale of 0–10 was your strongest experienced pain? (no pain) (excruciating pain) 0 1 2 3 4 5 6 7 8 9 10

9. In the past 6 months, what is the average value of the intensity of pain experienced on a scale of 0–10? (no pain) (excruciating pain) 0 1 2 3 4 5 6 7 8 9 10

10. In the last 6 months, how many days did you miss work or school because of pain in the face? ....days

11. In the past 6 months, how much did pain interfere with your daily activities, expressed on a scale of 0–10? (no interference) (impossibility to perform activities) 0 1 2 3 4 5 6 7 8 9 10

12. In the last 6 months, how much have your opportunities to participate in social and family life changed due to pain, expressed on a scale of 0–10? (no changes) (major changes) 0 1 2 3 4 5 6 7 8 9 10

13. How much did the presence of pain affect your ability to work in the last 6 months (including household chores), expressed on a scale of 0–10? (no changes) (major changes) 0 1 2 3 4 5 6 7 8 9 10

14a. Has it ever happened that you could not open your mouth all the way, i.e., have you had the feeling that your jaw was "locked" in some position? No..................................0 Yes..................................1

14b. Was the limitation of mouth opening so pronounced that it prevented you from eating? No..................................0 Yes..................................1

15a. Do you hear a popping sound when you open or close your mouth or when you yawn? No..................................0 Yes..................................

15b. Do you hear a grinding noise when you open or close your mouth or when you yawn? No..................................0 Yes..................................1

15c. Were you told about the sound(s), or did you notice yourself?

The primary outcome of pain is calculated based on questions 7–9. The average of the three ratings was multiplied by 10 to obtain a characteristic pain intensity score for the RDC/TMD ranging from 0 to 100.

Supplementary 2. OHIP-14

Every question is answered from 0 to 4:
not at all …………..0
rarely…………….. .1
occasionally............ 2
often……………....3
very often………....4

1. Functional Limitation

1. Have you ever had difficulty pronouncing words/sentences because of problems in the oral cavity?

2. Have you ever felt unable to taste well because of problems in the oral cavity?

2. Physical Pain

3. Have you ever had pain in your mouth?

4. Have you ever felt uncomfortable when chewing because of problems in the oral cavity?

3.Psychological Discomfort

5. Have you ever felt worried/anxious because of problems in the cavity?

6. Have you ever felt tense because of problems in the oral cavity?

4. Physical Disability

7. Have you ever felt dissatisfied with the food you consumed because of problems in the oral cavity?

8. Have you ever had to stop suddenly while chewing food because of problems in the oral cavity?

5.Psychological Disability

9. Have you ever had difficulty feeling relaxed because of problems in the oral cavity?

10. Have you ever felt embarrassed because of problems in the oral cavity?

6. Social Disability

11. Have you ever become irritable because of problems in the oral cavity?

12. Have you ever had difficulty carrying out your daily activities because of problems in the oral cavity?

7. Handicap

13. Have you ever felt that your life is unsatisfactory because of problems in the oral cavity?

14. Have you ever found it difficult to do things because of problems in the oral cavity?
